# A mixed-methods first-year evaluation of implementation outcomes for a bottom-up osteoarthritis management program across healthcare settings in Ireland

**DOI:** 10.1093/tbm/ibag017

**Published:** 2026-04-15

**Authors:** Clodagh M Toomey, Avantika Bhardwaj, Norelee Kennedy, Anne MacFarlane, The IMPACT (Implementation of osteoarthritis clinical guidelines together) Steering Committee, Stacey Grealis, Jacqui Browne, Bronwen Maher, Martina Blake, Peter Hayes, Adrian Gheiti, John O’Hora, Darragh Maguire, Ian Dowling, Brenda Monaghan, Helen French, James Young

**Affiliations:** School of Allied Health, University of Limerick, Limerick V94 T9PX, Ireland; Health Research Institute, University of Limerick, Limerick V94 T9PX, Ireland; Particpatory Health Research Unit, University of Limerick, Limerick V94 T9PX, Ireland; Centre for Implementation Research at UL, University of Limerick V94 T9PX, Ireland; School of Allied Health, University of Limerick, Limerick V94 T9PX, Ireland; Health Research Institute, University of Limerick, Limerick V94 T9PX, Ireland; Centre for Implementation Research at UL, University of Limerick V94 T9PX, Ireland; School of Allied Health, University of Limerick, Limerick V94 T9PX, Ireland; Health Research Institute, University of Limerick, Limerick V94 T9PX, Ireland; Health Research Institute, University of Limerick, Limerick V94 T9PX, Ireland; Particpatory Health Research Unit, University of Limerick, Limerick V94 T9PX, Ireland; School of Medicine, University of Limerick, Limerick V94 T9PX, Ireland

**Keywords:** implementation, osteoarthritis, equity, co-design, exercise, education

## Abstract

**Background:**

The Good Life with osteoArthritis Denmark (GLA:D®) non-profit initiative is a bottom-up approach to deliver evidence-based care, including exercise and education, to people with hip or knee osteoarthritis. GLA:D® was adapted for public and private healthcare in Ireland, using a participatory approach to co-design implementation strategies that would ensure optimal and equitable access.

**Purpose:**

To evaluate implementation outcomes of acceptability, adoption, appropriateness, cost, feasibility, fidelity, penetration and sustainability of GLA:D® Ireland across healthcare settings, in the first year.

**Methods:**

Mixed methods explanatory sequential design. GLA:D® training was advertised to physiotherapists in different healthcare settings around Ireland. Quantitative implementation outcomes were collected from the GLA:D® Ireland Registry. Semi-structured interviews were conducted with trained physiotherapists and participants and analyzed to identify themes.

**Results:**

In the first year, 32% of the 71 physiotherapists trained implemented GLA:D® in practice (*n* = 10 primary care, *n* = 2 public hospital, *n* = 3 private). Of 146 participants, 41% were from three sites with ≥1 physiotherapist trained. In interviews (10 physiotherapists, 9 participants), satisfaction with the structured nature that allowed individualization and self-management was reported. Physiotherapists used the program to tackle long waitlists and participants regained confidence to exercise. While equipment costs were low, more staffing and space were mediators for feasibility and sustainability across settings.

**Conclusions:**

While a high proportion of GLA:D® adopters were in primary care settings, penetration was more successful in public hospitals, with more resources and physiotherapists trained per site. Results suggest that implementation strategies could be adapted to incentivize training of multiple staff at one site and training of support staff. Top-down funding would help to ensure timely and equitable access to the program across healthcare settings.

## Graphical abstract

**Graphical Abstract:**
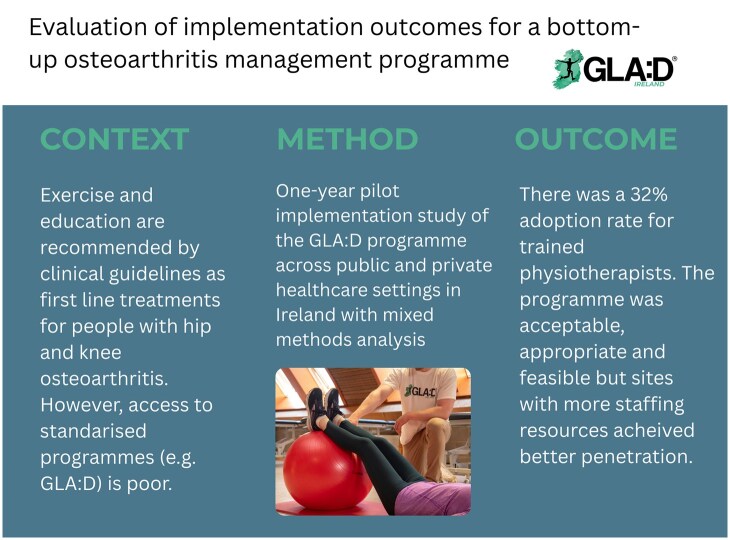

## Teaser

An evidence-based self-management program for osteoarthritis consisting of 8-weeks of exercise and education, was adopted by public and private healthcare settings in Ireland using a bottom-up approach.

Implications **Practice:** Group delivery of osteoarthritis management programs (OAMPs) like GLA:D® require flexibility from physiotherapists and managers but are considered acceptable, appropriate, feasible, with high fidelity and low cost across health settings where implemented in Ireland. Tailored implementation strategies for public and private healthcare settings are now needed to improve adoption, penetration and sustainability of such evidence-based services.

**Policy:** Staff shortages impede implementation of OAMPs and staff should be incentivized to prioritize group-based high-value care for chronic condition such as osteoarthritis. Healthcare insurance reimbursement models for OAMPs will help to make high-value care more equitable for patients.

**Research:** Implementation strategies should be co-adapted with researchers, clinicians, patients and policy-makers, and evaluated to ensure long-term sustainability and cost-effectiveness of these programs as part of healthcare delivery.

## Introduction

Osteoarthritis (OA) is a chronic lifestyle-related whole-joint disease, dominated by pain and limited function. While there are no current disease-modifying drugs for OA [1], symptom management is extremely amenable to exercise and self-management approaches, to limit the progression and impact of the disease on the individual [2]. However, implementation of these core recommended treatments in clinical practice, both globally and nationally, has been poor [3]. A failure to provide opportunities for people with OA to access this care has dire consequences, given 20%–30% of the adult population have the disease, given the economic burden and cost of increased surgical rates and a rapidly ageing population with reduced function and quality of life [4, 5]. In addition, it is well recognized that the prevalence of OA is on the rise, exacerbated by higher rates of obesity, sedentary behaviors and ageing populations [4].

Strategies to mobilize best evidence into high-value clinical care for OA require a pragmatic approach at all levels of care delivery [6], to ensure access of recommended care is equitable regardless of ability to pay or location. Implementation science methodologies can help in this regard, from the process of evaluating the barriers and facilitators to implementation, through tailoring strategies to improve implementation, and rigorously evaluating the effectiveness of these strategies. OA management programs (OAMPs) aim to deliver coordinated, evidence-based and non-surgical care for people with OA [7]. However, there are many proposed areas where optimal delivery of this high-value care can fall short. With regards to patient-level barriers, wait times, pain and other symptoms, low expectations or motivation have been cited as reasons for not engaging with OAMPs [8]. At the service-level, lack of resources and staffing or appropriate pathways of care are areas that need to be addressed [9, 10]. At the policy-level, a lack of prioritized funding for OAMPs is a major barrier to implementing these types of initiatives [6]. Implementation strategies that address all three levels are required.

The Good Life with osteoArthritis Denmark (GLA:D®) non-profit initiative is an example of an OAMP that was cross-culturally adapted for implementation in Ireland in October 2021 [11]. Available across different continents, countries, and healthcare settings, GLA:D® is a bottom-up approach to deliver physiotherapist-supervised evidence-based exercise and education programs to people with hip or knee OA, thus improving access and patient-reported outcomes [12]. However, research shows that introducing an intervention into a complex healthcare system is not enough to achieve uptake and sustainable change alone [13]. Irish healthcare is based on a two-tier system, whereby the private system offers faster access to and better choice of services for those who can afford insurance (about 47%) or to pay out of pocket [14]. Based on income or other needs, approximately 48% of the population have access to GP or medical cards, which provide access to essential public services free of charge, albeit with longer wait times [14].This can create large health disparities, with 90% of Irish individuals in the highest income quintile feeling “in good health” compared to 66% of those in the lowest quintile [14]. To address the needs of the population, patient-, service- and policy-level barriers need to be addressed for all settings and types of healthcare access.

There is a growing recognition that decisions regarding research related to healthcare services are best made collaboratively or by cocreation, involving all stakeholders at the table, and at each stage of the implementation process [15–17]. A participatory health research approach that incorporates views of researchers, clinicians and patients is a means to adapt an intervention and tailor implementation strategies to a given context, ensuring optimal and equitable access to GLA:D® in Ireland [11]. To understand the effectiveness of implementation strategies and efforts, the Implementation Outcomes Framework (IOF), developed by Proctor *et al* [18] offers a taxonomy to thoroughly conceptualize and measure effectiveness of implementation. While other countries implementing GLA:D® have reported primarily on feasibility [19, 20] and some additional outcomes using the RE-AIM QuEST Framework [21–23], a focus on all outcomes across public and private healthcare settings has not been conducted before. The objective of this study is to use a mixed-methods approach to evaluate the implementation outcomes of acceptability, adoption, appropriateness, cost, feasibility, fidelity, penetration and sustainability of GLA:D® Ireland across different healthcare settings, in the first year of implementation. This evaluation will allow further tailoring of implementation strategies for sustainability of the program.

## Methodology

### Study design

This study is reporting on a mixed methods evaluation of implementation outcomes from a hybrid effectiveness-implementation trial design (type III) [24], which is stage two of the larger IMPACT (IMPlementation of osteoArthritis Clinical guidelines Together) project [11]. The IMPACT project uses a participatory health research approach as described previously [11], to co-design implementation strategies (Stage 1) to embed an evidence-based program for hip and knee osteoarthritis (GLA:D®) in the Irish healthcare system, across public and private settings. Stage two (the current study) is focused on piloting and evaluating the implementation strategies, while Stage 3 will go on to adapt the strategies where needed, for nationwide rollout of the evidence-based program [11]. Quantitative registry data allowed for a broad understanding of implementation outcomes while qualitative interviews provided more rich, contextual information needed to move to the next stage of the project.

Since this was a pilot implementation study, no data was available to estimate the required sample size. This study was approved by multiple regional ethics committees, including Galway Clinical Research Ethics Committee (REC) (C.A. 2685), University of Limerick EHS REC (2020-12-13-EHS), National Orthopaedic Hospital Cappagh (NOHC-2022-ETH-MB-CEO- 331), and Clinical REC of the Cork Teaching Hospitals (ECM 4 (v) 16/11/2021 & ECM 3 (cc) 22/02/2022). Funding for this project was from an Emerging Investigator Award grant from the Health Research Board in Ireland (EIA-2019-008) and reporting guidelines were followed using the Good Reporting of A Mixed Methods Study (GRAMMS) Checklist [25].

### Co-design of implementation strategies

A steering committee of 14 stakeholders, representing researchers, physiotherapists in different healthcare settings, people with OA, a patient advocacy group, general practitioner and orthopedic consultant surgeon met online four times prior to initiation of the pilot, to co-design implementation strategies [11]. As described previously [11], this process was guided by an analysis of beliefs and implementation determinants using the local context, implementation theory (Consolidated Framework for Implementation Research (CFIR) - Expert Recommendations for Implementing Change (ERIC) matching tool [26]), review of the literature, and expert consensus. An example of a multi-faceted co-designed strategy was the GLA:D® Ireland Physiotherapist Implementation Toolkit [11]. This online toolkit contains a detailed step-by-step description of how to implement GLA:D® in clinical practice, including program requirements for time, space, equipment, and data collection, materials to support referrals and advertising and literature-backed evidence to gain managerial and consultant support.

### GLA:D® intervention recruitment

Consistent with a bottom-up, pragmatic approach to implementation, a sample of physiotherapists in Ireland were recruited to take part in a 2-day GLA:D® training course through typical methods of continuous professional development advertising (e.g. social media, Irish Society of Chartered Physiotherapists website, word-of-mouth). While training is the first step to implementing GLA:D®, a commitment to initiating the program was not a requirement for participation in training. Physiotherapists working across private and public healthcare settings and regions were trained to deliver the program between October 2021 and September 2022. The GLA:D® intervention consists of two to three education sessions and 12 (twice weekly) group neuromuscular exercise sessions as described in detail previously [27]. Additional training and discussion in the courses was dedicated to overcoming barriers to implementation that had been identified.

### Participants and setting

GLA:D®-trained physiotherapists who went on to deliver the GLA:D® intervention as part of their service in a public or private setting for patients with hip or knee OA were recognized as adopters of GLA:D® (GLA:D physiotherapists). People with OA who were registered on a GLA:D® program by their physiotherapist were recognized as participants of the program. Inclusion criteria were ≥ 18 years old and having joint problems from knee and/or hip that have resulted in contact with the healthcare system. Exclusion criteria were non-OA cause of symptoms (e.g. tumor), or other symptoms that are more pronounced than the OA problems (e.g. fibromyalgia, acute knee trauma in the past 6 months), or a lack of English language proficiency. Both GLA:D physiotherapists and participants were recruited via email for qualitative interviewing via maximum variation sampling to achieve representation based on sex, healthcare setting (all), and condition/ duration of pain (participants only) [28].

### Data collection

A core set of implementation outcomes as defined in the IOF [18] were evaluated in this study including: acceptability, adoption, appropriateness, cost, feasibility, fidelity, penetration and sustainability (Table 1). The evaluation was performed using an explanatory sequential design [29], whereby quantitative data were collected using the GLA:D® registry, followed by semi-structured interviews to understand implementation outcomes in more detail. The specific quantitative and qualitative source or outcome measure used to determine each outcome in the first year of implementation is defined in Table 1. For example, “adoption” was defined as the proportion of trained physiotherapists implementing GLA:D® from the convenience sample of physiotherapists who enrolled in the training. More contextual information on adoption, for example, the barriers encountered while initiating the program after training, were explored in semi-structured interviews. Written consent was obtained from all participants prior to data collection.

**Table 1:** Mixed methods analysis of GLA:D Ireland pilot using implementation outcomes framework [18].

Outcome	Definition	Quantitative data collection	Qualitative data collection
Adoption	Uptake and utilization of the program	Proportion of trained physiotherapists implementing GLA:D (i.e. registering patients) and participant registrations from private and public health settings across Ireland.	Interviews with physiotherapists and participants: Reasons/motivations/barriers to running or partaking in a GLA:D program
Acceptability	Satisfaction with various aspects of the program	Acceptability of intervention measure (AIM) (Post-course Physiotherapists); Proportion of GLA:D Physiotherapists satisfied with course; Proportion of participants satisfied with GLA:D program.	Interview with physiotherapists and participants: “Were you satisfied with the program?”
Appropriateness	Perceived fit, relevance, or compatibility of the program for a given setting, provider, or consumer; and/or to address a particular issue or problem	Intervention appropriateness measure (IAM) (Post-course physiotherapists).	Interview with physiotherapists: “How compatible is the GLA:D program in your setting?” Interview with physiotherapists and participants: “Does the program address a particular issue or problem?”
Cost	The financial impact of the implementation	N/A	Interview with physiotherapists and participants: “Have there been any cost implications?”
Feasibility	The extent to which the program can be successfully used or carried out within a given setting	Feasibility of intervention measure (FIM) (Post-course physiotherapists).	Interview with physiotherapists: “What challenges were encountered providing the program?” (related to setting and delivery)
Fidelity	The degree to which the program is implemented as it was prescribed in the original protocol	Deviation from recommended delivery format from registry.	Interview with physiotherapists: “Did you make specific changes to the original program? What did you change?”
Penetration	The integration of a practice within a service setting and its subsystems	Number screened during the study period; number completing baseline and follow-up questionnaires; Number and type of referral sources.	Interview with physiotherapists: “If a clinic manager were to ask you about implementing the program, what advice would you offer?” Interview with participants: “Would you recommend this program to other people with osteoarthritis and if so, what advice would you give them?”
Sustainability	The extent to which the implemented program is maintained	N/A	Interview with physiotherapists: “Do you intend to continue delivering the program?” Interview with participants: “Do you intend to continue doing the exercises?”

### Quantitative data collection

Quantitative implementation outcomes (Table 1) collected from the GLA:D® Ireland Registry from the first year (programs implemented between November 2021 and 2022) were extracted for this analysis. The registry allows data to be collected after a physiotherapist completes GLA:D® training (satisfaction with training, intention to deliver a program), when the physiotherapist registers a participant (contact details, referral source, method of program delivery), and during baseline and follow-ups (patient reported outcome measures). Registry data were cleaned upon extraction to check for incomplete entries but were not precluded from the current analysis on the basis of having attempted but not completed all of the questionnaire. After training, the physiotherapists also completed a questionnaire including three short outcome measures related to implementation, collected using the registry: Acceptability of Intervention Measure (AIM), Intervention Appropriateness Measure (IAM) and the Feasibility of Intervention Measure (FIM). These validated and reliable items have scale values ranging from 1 to 5, with higher scores indicating greater acceptability, appropriateness, or feasibility [30]. Physiotherapists were also asked about how they intend to use the GLA:D® Ireland course content in this questionnaire. All data were collected and stored securely using the REDCap™ electronic data capture tool hosted at the University of Limerick [31].

### Qualitative data collection

Semi-structured interviews were conducted with GLA:D® physiotherapists after implementation of their first program and with participants after they had completed a program. Interviews were dual purpose: to answer questions related to barriers and enablers to uptake [32] and evaluate implementation outcomes. Therefore, the topic guide was designed for both purposes, guided by the CFIR and previous literature [22]. (Supplementary File 1) Questions aligning to specific outcomes are stated in Table 1. Interviews were conducted by a PhD candidate [AB] with training in qualitative methodologies who had no prior interactions with the participants. Interviews were conducted via telephone or online (Microsoft Teams, Microsoft Corporation). The interviews were audio-recorded and transcribed verbatim by AB, with any personal identifiers removed.

### Data analysis

For quantitative analysis, outcomes were analyzed descriptively using frequencies/proportions for categorical variables and means and standard deviations or medians and interquartile ranges for continuous variables. Qualitative analysis first involved reflexive assessment of pre-existing perspectives and biases by CT (clinician-scientist with 15 years’ experience) and AB, using field notes to document decisions. Qualitative implementation outcomes were analyzed deductively by one author (CT) using interview transcripts. After multiple readings, the pre-defined outcomes were assigned from units of the text and then inductively grouped into similar themes using NVivo software (QSR International). A random sample of a third of the interviews were analyzed independently by a second author (AB) with results compared and discussed to resolve any discrepancies. A final collaborative review of themes was undertaken once all analyses were complete and presented to the project steering committee for further interpretation of combined data.

## Results

### Sample

Demographic details for all physiotherapists and participants are presented in Table 2. Interviews with GLA:D® physiotherapists and participants were conducted within six months of having completed the pilot programs. There was a response rate of 50% (*n* = 10) of GLA:D® physiotherapists and 26% (*n* = 9) of participants contacted for interview. Interviews ranged in duration from 22 to 54 minutes.

**Table 2:** Demographic characteristics of study sample.

	GLA:D® non-adopters *n* (%)	GLA:D® adopters *n* (%)		Qualitative sub-sample *n* (%)	
	Physiotherapists, *n* = 48	Physiotherapists, *n* = 23	Participants, *n* = 114	Physiotherapists, *n* = 10	Participants, *n* = 9
**Age**					
40-49	–	–	5 (4.4)	–	–
50-59	–	–	29 (25.7)	–	2 (22.2)
60-69	–	–	42 (37.2)	–	5 (55.6)
70-79	–	–	31 (27.4)	–	2 (22.2)
80-89	–	–	6 (5.3)	–	–
**Gender**					
Female	35 (72.9)	17 (73.9)	88 (77.2)	6 (60)	7 (77.8)
Male	13 (27.1)	6 (26.1)	26 (22.8)	4 (40)	2 (22.2)
**Province in Ireland**					
Munster	23 (47.9)	10 (43.5)	37 (32.5)	3 (30)	4 (44.4)
Ulster	1 (2.1)	2 (8.7)	10 (8.8)	2 (20)	–
Leinster	16 (33.3)	6 (26.0)	33 (28.9)	1 (10)	5 (55.6)
Connacht	8 (16.6)	5 (21.7)	34 (29.8)	4 (40)	–
**Healthcare setting**					
Public	36 (75.0)	20 (87.0)	94 (82.5)	8 (80)	4 (44.4)
Private	12 (25.0)	3 (13.0)	20 (17.5)	2 (20)	5 (55.6)
**Location**					
City	16 (33.3)	16 (69.6)	81 (71.0)	7 (70.0)	6 (66.7)
Urban	16 (33.3)	4 (17.4)	14 (12.3)	0	0
Urban/Rural	16 (33.3)	3 (13.0)	19 (16.7)	3 (30.0)	3 (33.3)

“- ”: not applicable.

#### Adoption

In the first year of GLA:D®, 71 physiotherapists attended one of three training courses (41% primary care, 38% public hospital, 21% private). In response to the post-training question of “How do you intend to use the GLA:D® Ireland course content?”, 38% responded that they planned to provide GLA:D® to patients within 3 months, 44% responded that they planned to provide GLA:D® to patients within 6 months, and 18% were going to integrate learnings into their current service as it was not feasible to run group programs. There were 23 (32%) physiotherapists who implemented GLA:D® across 15 distinct sites (*n* = 10 primary care, *n* = 2 public hospital, *n* = 3 private) and across all four provinces in Ireland, between November 2021 and 2022. Adopters of GLA:D® were more likely to practice in a city location compared to urban or urban/rural mix (Table 2). In the 6-month period after the last training course, there were no additional physiotherapists who implemented a program. In this time, 146 participants with OA were registered and n=114 (78%) submitted baseline questionnaires (Table 2).

Four themes were drawn from qualitative interviews related to *adoption*. The first, *“Time Commitment and Prioritisation”*, acknowledged the initial challenge and organization required by physiotherapists to get the program off the ground, particularly in primary care roles with many competing priorities and time demands.

*PT1 (Primary Care Physiotherapist, Female): “But it took more organization than maybe I initially thought it would, and specially from the testing and the patients and the admin side of it and but if I was to do it again, it obviously would be a lot easier because I know the process now”*

*“Finding an Appropriate Venue”* was the second theme related to *adoption*. While not all clinics or hospital settings had an appropriate space to run an exercise class, there was a large pay-off to the effort in locating an off-site community space, to “de-medicalise” the treatment and improve visibility.

*PT10 (Public Hospital Physiotherapist, Male): “…one of the participants…said something along the lines of like when I’m going to hospital, I feel like I’m unwell. Whereas when I’m going to the gym, I feel like I’m going there to exercise, it’s something kind of positive I’m doing for my health.”*

The theme: *“Communicating with Managers”* alluded to the benefit of having managerial or colleague support to run the programme but acknowledged that having the research element as an integral part of GLA:D® made it an easier conversation to have, aligning with current healthcare policy aims in Ireland for universal, patient-centred care (i.e. Sláintecare [33]).

*PT5 (Public Hospital Physiotherapist, Female): “…if I was looking at talking to my manager and commissioning services, I’d say this is a high value treatment, that’s patient-centred. And if it’s delivered in the community, then it is within the vision of Sláintecare. This is exactly the kind of care we should be delivering because it is high value. And that’s how I’d make the case. And if the research is there to back it up.”*

Finally, for *“Advantage of GLA:D Resources”,* physiotherapists found the evidence-based GLA:D® slides, handouts, resources and structured framework made it easier to get a programme running, with less doubt about the effect it was going to have.

*PT5 (Public Hospital Physiotherapist, Female): “I think the fact that you get the resources sent out as well, that cuts out work that can sometimes make it difficult to get these kind of things off the ground.”*

#### Acceptability

Following the GLA:D® training, *acceptability* was highly rated by physiotherapists using the AIM outcome measure (mean 4.7, SD 0.5, range 4–5). In addition, 100% of physiotherapists agreed or strongly agreed that they were satisfied with the training. After program delivery, 93% of participants were satisfied or very satisfied with GLA:D®. In interviews, two themes related to *acceptability*, included “*Satisfaction with Structured Approach”* and *“Positive Social Environment”.* All stakeholders were clearly satisfied with having a structured exercise and education program to follow, with flexibility of progressions and regressions for each individual, planned dates in the diary for attendance and detailed educational materials.

*PT5 (Public Hospital Physiotherapist, Female): “But I’m just glad that it is standardised so that in the future, if I’m sitting in a clinic, in a triage clinic and a patient with knee OA, and I send them to the GLA:D community program, I know exactly what they’re going to get, and I can tell the patient exactly what they’re going to get. And I think that’s the benefit of the training.”*

The group element of GLA:D® was seen as positive with regards peer-learning, motivation, adherence, social support, building self-efficacy and comradery.

*PwOA4 (Private Patient, Female, Hip OA): “And also, you’re listening to the other women and you kind of realise they’re going through the same kind of thing that you are, and they’re suffering. They’re not having great days either, because you can kind of maybe initially feel as disillusioned or what’s wrong with my body?”*

#### Appropriateness

Following the GLA:D® training, the mean IAM as rated by physiotherapists was 4.5 (SD 0.5) with a range of 3-5. Under the theme, “*Accessibility”,* physiotherapists described the program as accessible to all abilities, compatible and catering very well to the needs of their patients. They were very satisfied to be able to offer an evidence-based service to help manage long waitlists for subacute or chronic conditions. Participants felt stronger and that the program has kept them out of surgery.

*PT7 (Primary Care Physiotherapist, Female): “…two of my patients took themselves off the orthopaedic waiting list. I know that. I mean that’s serving their needs as well.”*

There was a strong sense that the exercise and self-management components of the program were *“Empowering the Patients”.* Participants learned why they needed to exercise and set their expectations for the class and the progression of their condition.

*PT6 (Private Physiotherapist, Male): “I think it empowers them through the education component… to give a bit more confidence in terms of what they can do as opposed to the fear of what they shouldn’t do.”*

*PwOA2 (Public Hospital Patient, Female, Knee OA): “And it gave me the confidence…to be able to bend the knee more and push it, not to be precious with the knee because it was able to withstand much more once…the muscle around it had developed a bit better.”*

This was also linked with *“Tackling Misconceptions”* around OA and using what they had learned after partaking in the programme. Participants spoke of being more knowledgeable on the most appropriate hierarchy of treatment, what to do during a flare-up, and the importance and safety of mobility and activity.

*PwOA6 (Private Patient, Female, Knee OA): “Well, it was interesting to learn about the arthritis and things that I would have had preconception that maybe everything needed to be MRI’d and then diagnostically then treated (surgery), whereas the GLA:D program indicated that that’s not necessarily true.”*

#### Cost

Some physiotherapists felt that there were *“Low-level Costs”* incurred in running the program from an equipment standpoint, but these were once-off costs for equipment that were mostly already available in the clinic. The main cost incurred was time to organize and deliver the 7-week program, with some lack of clarity regarding whether programs would be reimbursed in future for private patients. Some private physiotherapists reported running a no- or low-cost program as a pilot to get used to implementing their first program.

Physiotherapists highlighted the availability of *“External Funding”* for equipment or space rental that was available in their organization or community (e.g. Sports Partnerships).

*PT5 (Public Hospital Physiotherapist, Female): “The rent is not expensive, but it’s whether or not the hospital would be happy to pay for that. And that’s the conversation I actually have to present, I have to talk to the CEO.”*

#### Feasibility

Following the GLA:D® training, the mean FIM as rated by physiotherapists was 4.1 (SD 0.6) with a range of 3-5.

A theme regarding frequency of classes taking up much *“Clinical Time”* was brought forward by physiotherapists. Strategies to make best use of time included running two classes back-to-back to reduce set-up or travel time, getting assistance from clinical assistants or students with set-up and equipment, and screening participants on the phone.

*PT8 (Primary Care Physiotherapist, Female): “It’s a really good program, but it’s a lot of time consumed once you’re in the sessions. It takes a lot of time over a number of weeks, and two sessions a week is a lot.”*

While some physiotherapists did not have additional support from colleagues, *“Personnel Resources”* was a key theme for *feasibility*. Administrative support, physiotherapy assistants or students were all highlighted as key enablers to delivery of the program.

Overall, and with experience, GLA:D® was deemed to be feasible within the practice.

*PT6 (Private Physiotherapist, Male): “I think with GLA:D you’re constantly learning or changing and there’s that flexibility within the system to modify and progress it as you feel will work best for you, your clinic, and your patients.”*

#### Fidelity

With regards *“Modifications”,* physiotherapists reported not having changed the program from how they were trained to deliver it. Many adapted and individualized exercises beyond prescribed GLA:D® exercise “levels”, which is within the scope of the program.

*PT2 (Primary Care Physiotherapist, Female): “if they didn’t have a mat or a ball, we discussed alternative ways of doing it at home, so you get a similar exercise.”*

Physiotherapists and PwOA reported *“High Adherence”* to the program. This often came as a surprise to physiotherapists who were experienced in exercise class delivery and put this down to the education intervention scheduled before the exercises.

*PT5 (Public Hospital Physiotherapist, Female): “…they all said that it had made a difference to their pain and that they understood now that it was safe to exercise. So, I think the fact that they turned up and they adhered to the program speaks volumes. I mean, it’s very unusual to get 100% attendance in physiotherapy and yeah, they all turned up.”*

#### Penetration

There were 114 GLA:D® participants (78%) who completed their baseline questionnaire and *n* = 51 (39%) who completed the follow-up questionnaire of the 147 participants registered in the database. Of the participant registrations across 15 sites, 41% were from the three sites (*n* = 1 primary care, *n* = 2 public hospital) that had more than one physiotherapist trained. Referrals came primarily from other healthcare professionals (45% from GP or other doctor, 29% from other physiotherapist, 14% from orthopedic surgeon) and 10% were screened from an existing waitlist. Of those referred from another source, *n* = 29 (28%) were referred for GLA:D® specifically.

Both physiotherapists and participants described how a greater *“Awareness of GLA:D®”* could improve implementation. Word of mouth, research publications, and information resources for GPs and orthopedic teams were all cited as ways to increase penetration while the program becomes more established.

*PwOA2 (Public Hospital Patient, Female, Knee OA): “I’m so surprised that so many people don’t know about GLA:D… if it was known in the different hospitals that do orthopaedic surgery to give people the option of participating in that, I think more people might take it up.”*

*“Support of Colleagues and Referrers”* was a theme that highlighted how GLA:D® was more efficiently integrated within a service when it was driven by those with influence (managers, peers and consultants).

#### Sustainability

With regards *“Sustainability in Service”*, some physiotherapists considered the GLA:D® program to be established within their service, with 3-4 cohorts complete within one year. Smaller services were waiting on additional colleagues to be trained, managerial go-ahead or locating a more long-term community space before delivering another program. Other physiotherapists had moved roles or were in the middle of organizational changes that limited innovation.

For the theme of *“Continuing at Home”*, physiotherapists described how they were very confident that participants could continue to exercise at home after GLA:D® and that printed materials and sign-posting to local physical activity initiatives were enablers to achieving this. Participants described how GLA:D® motivated them to be more active and have a plan for continuation. This varied between performing the exercises at home, joining other exercise classes and increasing walking, cycling or gardening for most.

*PwOA9 (Primary Care Patient, Female, Hip OA): “And if I’m stiff in the morning, if I’m not able to do the full exercise programme, I just do the warm-up exercise and then do the programme later that day whenever I have the time to do it. So, yeah, I just feel it’s part of my life now to keep moving and it’s working.”*

While all intended on being more active, some preferred having scheduled classes to sustain motivation.

*PwOA6 (Private Patient, Female, Knee OA):: “But I would like if they had GLA:D programmes where you could sign up and you could go maybe once a week, because I do think it’s a fantastic programme but just from the motivational point of view, I find that I’m much better at doing stuff when I book into a class rather than trying to do it myself.”*

## Discussion

This study used a mixed methods approach to measure the Proctor implementation outcomes and understand the effectiveness of co-designed implementation strategies to embed an evidence-based OAMP across public and private healthcare systems in Ireland in its first year.

*Adoption* is one of the key implementation outcomes, used across many frameworks (e.g., IOF, RE-AIM, CFIR 2.0), and considered a priority in this context to build capacity for implementation of GLA:D® across health settings in Ireland. Quantitative registry findings indicated a 32% adoption rate across trained physiotherapists, which is comparable to other OAMPs globally, for example, the Better management of patients with OsteoArthritis (BOA) programme in Sweden reported a 33% adoption rate following training [34]. Other countries implementing GLA:D® reported intention to adopt or proportion of adopters in a follow-up survey rather than proportion of those trained so direct comparison is not possible [19, 21].

Interpretation of the adoption rate should consider that some physiotherapists (18%) reported attendance at training to embed elements of up-to-date, evidence-based care into their service, rather than with the intention to implement a group program. It is also possible that some physiotherapists adopt GLA:D® but do not register participants in the database. It appears that the intention to deliver a program within a 6-month period was very high (82%) but this did not translate into adopting the program when clinicians returned to their practice. Interviews with GLA:D® adopters provided some context in this regard, mainly from primary care physiotherapists experiencing difficulty with prioritizing time over other roles and acute conditions, and others who had to initially locate a space to run the program. Being supported by managers in their endeavors and having the GLA:D® resources and evidence helped them to adopt the program. Given the large proportion of public sector clinicians trained in Ireland (80%) compared to other some countries where GLA:D® implementation has been evaluated (20% in Australia [21]and 46% in Switzerland [19]), these barriers require more thorough scrutiny and tailored strategy design or adaptation. On the other hand, difficulties with adoption for private practice clinicians was also evident as only 3/15 had implemented a program and had provided their first program free of charge or low cost. Consistent with challenges experienced in other countries [21, 35], changing private healthcare insurance reimbursement models to support access to OAMPs is a necessary step to ensure equitable high-value care is in place. With mounting evidence to suggest surgery prevention and ensuing long-term healthcare savings will increase with the availability of GLA:D® [36, 37], this multi-faceted approach is urgently needed.

*Acceptability, appropriateness* and *feasibility* are sometimes considered antecedent assessments to the success of *adoption* [38]. The AIM, IAM and FIM, measured directly after GLA:D® training, were all scored highly by physiotherapists. Similar to GLA:D® programs delivered in Australia, Denmark and Canada, participant satisfaction rates were over 90% [20, 22, 23]. Those who delivered or took part in the program were resoundingly positive about the experience, particularly with regards to appropriateness of the combination of exercise with education, and the flexibility to adapt the content to the individual or group. Across all OAMPs measured in randomized control trials, education interventions have been found to be most effective for pain and function when delivered in combination with exercise therapy [39]. As an integral part of GLA:D®, physiotherapists were appreciative of standardized resources to help deliver this component, and believed timing education delivery before exercise was important for quelling misconceptions about exercise and improving adherence to the program. As a core component of clinical guidelines for OA [40], it is important that clinicians and funders do not under-value the role that standardized education plays in improving behaviors and outcomes. This, in combination with the social support offered by peers during the supervised sessions, were attributable features of the success of the program across the different health settings. Overcoming initial barriers to feasibility and clinical time and optimizing OAMPs in this way is in line with recent calls to integrate group-mediated programs into routine care to tackle the increasing burden of chronic conditions in our ageing population [41, 42].

Only two public hospitals implemented GLA:D® in the first year but were much more successful with *Penetration* in terms of number of registered participants and consistent referrals, due to increased staffing resources and geographical pool. Since public hospitals tend to be located in cities, the data suggests that more strategies could be put in place to ensure availability of programs is consistent across urban and rural locations in Ireland. Physiotherapists who were the sole GLA:D®-trained provider at a site reported difficulty adopting and maintaining the program without additional colleague support. Since many private practice physiotherapists in Ireland tend to operate in small clinics with 1-3 staff members, this may be a common barrier featured across public and private settings. As a bottom-up initiative with limited resources for advertising, GLA:D® is dependent on word-of-mouth to drive demand for the program through clinical practice networks and patient advocacy groups. While use of a “mass media” campaign is an unlikely strategy for a non-profit initiative [26], physiotherapists and participants gave suggestions to increase awareness and buy-in of GLA:D® in Ireland through providing information to referring GPs and lead consultants across regions.

Consistent delivery of GLA:D® will be necessary for *sustainability* of the program, which may be difficult to achieve where staff shortages exist or staff turnover is high. In most other GLA:D® countries, initial implementation occurred primarily in the private sector [19, 21, 22, 27], with later efforts to successfully adopt the program in public settings [23]. Targeted strategies to improve implementation in the public sector in Ireland were deemed to be successful since GLA:D® was adopted primarily in public health settings (80%), with higher adoption rates for public vs. private physiotherapists (36% vs. 20%). This may lead to more equitable access of value-based care for people with OA who are not in a position to pay. However, further research is required to evaluate access based on sociodemographic profiles to determine if additional barriers exist, as per recommendations to address health disparities in implementation [43]. Additionally, longer periods of observation will explore if *sustainability* of the program differs by healthcare setting, in terms of continued program offering and factors related to participant program adherence. It was apparent that there was some patient demand for weekly continued supervision or drop-in/booster sessions to improve long-term behavior change, as described by GLA:D® “graduates” in other countries [44]. As reported in other OAMPs [45], utilizing community partnerships such as local sports partnerships were highlighted as important by GLA:D® physiotherapists for both locating community spaces to deliver the program and ensuring effective transition to community physical activity initiatives after the program.

This study is strengthened by its mixed methods approach, utilizing registry data for a high-level analysis, combined with qualitative interviews for more theory-driven (CFIR) contextual interpretations of implementation outcomes. Interviews were conducted once physiotherapists and participants had completed a program to gain valuable reflections, rather than once initial training had been completed. While important information was gathered from those who had to overcome barriers to implement a program, this study would have benefitted from interviews with non-adopters.

The definition of adoption in this study was restricted to the proportion of physiotherapists who implemented a program after deciding to take part in the training. While this approach aligns with the pragmatic nature and bottom-up approach of this implementation study, we were not able to capture information on those who decided not to take part in the training (e.g. due to location, timing) or who were not aware of the training taking place. Future research should capture this data, and establish barriers to receiving the training and determine adoption rates using a regional top-down approach to mandate supervised evidence-based structured programs for people with OA when they first present in primary care.

While the interviewed participants were mostly representative of the larger samples, there was limited participation from private sector, thus increasing the likelihood of biased qualitative results. The response rate for interviews was low for participants (26%) and likely due to research burden having just completed the GLA:D program and associated data collection. Nonetheless, no new themes were being generated in later interviews suggesting data saturation. Since the private practice physiotherapists ran a low- or no-cost pilot program for participants, there was no difference in the interpretation of cost between private and public patients. However, in future this may become a barrier to participation as charges are introduced and as patients wait for access in the public sector. Long waiting lists are the primary cause of unmet medical needs in Ireland, with individuals in the lowest income quintile nearly three times more likely to report unmet needs due to delays compared to those in the highest quintile [14].

## Conclusions

While a high proportion of GLA:D® adopters were in primary care settings, penetration was more successful in public hospitals, with more resources and physiotherapists trained per site. Implementation in private practices was limited, requiring further inquiry with non-adopters. While the program was deemed to be acceptable, appropriate, feasible, with high fidelity and low cost across settings, results suggest that implementation strategies could be adapted to incentivize training of multiple staff at one site, facilitate training of support staff (e.g. administration staff, physiotherapy assistants), make reimbursement models transparent, and identify appropriate community spaces for delivery. These efforts, in addition to top-down funding of resources, may help to ensure timely and equitable access to the program for a high-burden joint disease that will be applicable across diverse health settings and regions.

## Supplementary Material

ibag017_Supplementary_Data

## Data Availability

De-identified data from this study will be made available in a public archive after publication of the research results.
