# Supplementary material for: A mixed-methods first-year evaluation of implementation outcomes for a bottom-up osteoarthritis management program across healthcare settings in Ireland
Source: Transl Behav Med. 2026 Apr 15;16(1):ibag017. doi: 10.1093/tbm/ibag017 (PMC13082370; doi:10.1093/tbm/ibag017)
Supplement: ibag017_Supplementary_Data [file ibag017_supplementary_data.docx]

**Supplementary File 1**

**1.1. Semi-structured interview guide for pilot physiotherapist interviews**

1. DELIVERY: How did you deliver the program? (i.e., face-to-face, online or combination)
2. OVERALL EXPERIENCE/BARRIERS: Please tell me about your experiences providing the education and exercise program to people with hip/knee OA.

Probes:

- - What did you like about the program?
  - What worked well?
  - Were you satisfied with the programme overall?
  - What challenges were encountered providing the program?
  - What would be your advice to others to avoid identified challenges?

(Cover exercise, education, supervision, group element, face to face or online)

Additional online probes:

- How do you think it compares to consulting with patients face to face?
- How did the online nature of the program affect your communication style and the methods you use to develop a relationship with your patients?

1. FACILITATORS: Is there anything you wish you had known that would have helped you in delivering the program?

Probes: consider asking about the following areas:

- - Training you received to deliver the program
  - Processes or operationalization of organizing program delivery
  - Processes or operationalization of patient database
  - Additional knowledge or supports to provide the patient education
  - Additional knowledge or supports to provide the patient exercise component

Additional online probes:

- Additional knowledge or resources to support online delivery

1. LOGISTICS AND FIDELITY: If other therapists were going to start providing the program, what advice would you give them and/or the clinic manager?

Probes:

- - What suggestions or recommendations would you offer related to educating and preparing the therapists to deliver the program?
  - What suggestions or recommendation would you provide concerning the logistics of program delivery for the facility?
  - Were there specific resource and cost implications?
- Did you make specific changes to the original programme? What changes did you make and why?

1. APPROPRIATENESS AND SUSTAINABILITY – Do you intend to continue delivering the programme?

- How compatible is the GLA:D programme in your setting?
- Does the programme help to address a particular issue or problem that was there?
- What would you need to help you to continue delivering the programme long term?

Additional online probes:

- What would your preference be for delivery (face to face or online)? Why?
- What advantages or disadvantages do you see that the online offers over in-person visits?
- How well do you think your patients understood the exercises and physical activity plan you prescribed?
- How confident were you that your patients could perform the exercises safely and effectively at home on their own?
- How confident were you that your patients would adhere to the exercise/activity programs?

1. Is there anything else you’d like to tell me about the program and its delivery?

**1.2 Semi-structured interview guide for patient interviews**

1. DELIVERY: How did you receive the program? (i.e., face-to-face, online or combination)
2. OVERALL EXPERIENCE/BARRIERS: Please tell me about your experiences in taking part in the GLA:D Ireland education and exercise programme.

Probes:

- - What did you like about the program?
  - What worked well?
  - Were you satisfied with the programme overall?
  - What challenges were encountered taking part the program?
  - What would be your advice to others to avoid identified challenges?

(Cover exercise, education, timing, supervision, group element, face to face or online, any changes in symptoms, surveys, any costs or accessibility issues)

Additional online probes:

- How do you think it compares to consulting with physiotherapists face to face?
- How did the online nature of the program affect your communication style and how you developed a relationship with your physiotherapists?
- Did you encounter any problems with setup for online classes (software, internet, space, equipment)? Did you need/get any extra assistance from your physiotherapist or family/friends?

1. FACILITATORS: Is there anything you wish you had known at the beginning of the programme that you did not know about?

Probes: consider asking about the following areas:

- - Participant information sheets and information from your physiotherapist
  - Time commitment to attend sessions and complete surveys
  - Additional knowledge or supports for patient education
  - Additional knowledge or supports for patient exercise component

Additional online probes:

- Were there any other supports that would have helped with the online programme?

1. APPROPRIATENESS AND SUSTAINABILITY – Do you intend to continue doing the exercises?

- Do you use the information that you have learned from the programme currently or plan to?
- Are you confident performing the exercises in your own home?
- Did the programme address a particular issue or problem that you had?
- Do you think you have increased your physical activity and are there any particular resources you have used to do this? (e.g. joined a walking group, sports club)
- Would you recommend this programme to other people with osteoarthritis and if so, what advice would you give them?

Additional online probes:

- How well do you think you understood the exercises and physical activity plan you were prescribed?
- How confident were you that you could perform the exercises safely and effectively at home on your own?
- How confident were you that you would adhere/stick to the exercise/activity programs?
- What would your preference be for delivery (face to face or online)? Why?
- What advantages or disadvantages do you see that the online offers over in-person visits?
- What advice would you give to people who are unsure about doing the programme online?

1. Is there anything else you’d like to tell me about the program and its delivery?
